# Preoperative Clear Fluid Fasting Duration and Arterial Hypotension During Anesthesia Induction: A Narrative Review

**DOI:** 10.3390/jcm14196950

**Published:** 2025-10-01

**Authors:** Filomena Di Vezza, Claudia Cacace, Marco Sanvitti, Federico Bilotta

**Affiliations:** 1 Department of Anesthesiology and Intensive Care, University of Tor Vergata, 00133 Rome, Italy; filomena.divezza@ptvonline.it (F.D.V.); claudia.cacace@ptvonline.it (C.C.); 2 Department of Pediatric Surgery, “Sapienza” University of Rome, 00185 Rome, Italy; sanvitti.1927256@studenti.uniroma1.it

**Keywords:** preoperative fasting, clear fluids, anesthesia induction, arterial hypotension, hemodynamic stability, ultrasound monitoring

## Abstract

**Background**: Preoperative clear fluid fasting is intended to reduce aspiration risk, but prolonged abstinence may impair hydration, comfort, and cardiovascular stability. Arterial hypotension during anesthesia induction is a common perioperative complication, and its association with fasting duration has become an important concern. The objective of this review was to evaluate the relationship between the duration of preoperative clear fluid fasting and the risk of arterial hypotension during anesthesia induction in both adult and pediatric populations. **Methods**: A structured PubMed search identified 17 studies, including randomized controlled trials, prospective cohorts, registry-based analyses, and interventional imaging investigations. Data were extracted on patient age, fasting duration, hypotension definitions, and monitoring modalities. Subgroups included adults, pediatric patients, and studies employing echocardiography or ultrasound to evaluate preload. **Results**: A total of 96,017 patients were included (77,978 adults; 17,685 children). In adults, fasting beyond two hours was associated with hypovolemia and a greater incidence of post-induction hypotension, while fasting of ≤2 h improved hemodynamic stability without increasing aspiration risk. Pediatric studies demonstrated fasting durations often exceeding 6–10 h, correlating with higher odds of hypotension and metabolic derangements. Liberalized regimens, including carbohydrate-containing fluids, were consistently safe. Ultrasound-based studies revealed increased inferior vena cava collapsibility and reduced ventricular filling after prolonged fasting, providing a mechanistic explanation for blood pressure instability. **Conclusions**: Prolonged preoperative fasting was not consistently an independent predictor of peri-induction hypotension in all populations; however, data from large adult and pediatric studies demonstrate that extended fasting increases hypotension risk through volume and metabolic depletion. These findings support the importance of liberalized fasting policies and proactive fluid optimization to reduce early hemodynamic instability during anesthesia induction.

## 1. Introduction

Clear fluids are those fluids which, when held to the light, are transparent, colorless, and free from solid or particles and include: water, tea, coffee, broth, ice chips, and certain juices like apple or white grape juice [1]. Access to clear fluid ingestion is proven to be safe 2 h before the procedure [1,2].

Pre-operative clear fluid fasting refers to the abstention from clear fluids for a defined period prior to anesthesia induction [1]. The primary objective of this practice is to reduce gastric content volume and acidity, thereby minimizing the risk of pulmonary aspiration during induction. Pre-operative clear fluid fasting for adults undergoing elective general anesthesia has remained a cornerstone of safe perioperative practice for almost a century [3], but the concept that the longer the fasting, the safer it is for the patient, has led to excessive pre-operative food and fluid restriction, with no benefits for the patients [4].

Arterial hypotension during anesthesia induction in adults is defined as a mean arterial pressure (MAP) below 60–70 mmHg or a systolic arterial pressure (SAP) below 100 mmHg [5], whereas in pediatric patients, thresholds are less standardized and often rely on age-specific blood pressure percentiles [6]. Arterial hypotension is a frequent perioperative event associated with increased risk of postoperative complications, including acute kidney injury, myocardial injury, and adverse neurological outcomes [7,8].

The American Society of Anesthesiologists (ASA) recommends that both adults and children may safely consume clear fluids up to 2 h before anesthesia [1]. The European Society of Anesthesiology and Intensive Care (ESAIC) issues similar recommendations, allowing clear fluids until 2 h preoperatively in both age groups [9]. Differences between these guidelines include the ESAIC’s explicit endorsement of carbohydrate-containing clear drinks in healthy patients, while the ASA guidelines are more conservative, noting they may be considered. Both societies agree that prolonged clear fluid fasting offers no additional protection and may impair patient comfort, hydration, and cardiovascular stability.

Several studies [4,10,11,12,13,14,15] suggest a possible relationship between preoperative clear fluid fasting and more pronounced arterial hypotension during anesthesia induction, but no dedicated and comprehensive studies report more recent clinical evidence.

The aim of this narrative review is to summarize the current evidence regarding the relationship between preoperative clear fluid fasting duration and arterial hypotension during anesthesia induction in adult and pediatric populations, and to report evidence extracted by echocardiographic and ultrasound monitoring performed at different intervals around anesthesia induction.

## 2. Materials and Methods

Prolonged clear-fluid fasting was evaluated through the analysis of 17 studies [4,10,11,12,13,14,15,16,17,18,19,20,21,22,23,24,25]. Three additional reviews were considered to provide context but did not contribute to the patient count [26,27,28]. The review was based on a structured PubMed search with keywords including “preoperative fasting,” “clear fluids,” “anesthesia induction,” “hemodynamics,” and “hypotension” combined using Boolean operators AND and OR to broaden and refine the search results. For each study, data were extracted on patient age group, fasting duration for clear fluids, definition of hypotension, blood pressure measurement methods, type of surgery, and sample size. Definitions of hypotension differed, with most adult trials using a mean arterial pressure threshold between 60 and 70 mmHg, while pediatric investigations more often relied on age-adjusted percentile values. Only studies in elective surgical settings were considered, and all specified clear fluid fasting intervals in accordance with, or in comparison to, ASA or ESAIC recommendations.

Further analysis was performed by categorizing the included studies into three subgroups: those conducted exclusively in adult populations [4,10,11,12,13,14,18,26], those focused solely on pediatric patients [16,17,20,22,23], and those studies in which echocardiographic parameters such as the inferior vena cava collapsibility index and left ventricular end-diastolic area were used to estimate pre-induction hypovolemia and its potential contribution to arterial hypotension [14,19,21,24,25].

## 3. Results

This review included 96,017 patients across 17 studies, of whom 77,978 were adults and 17,685 were children. The included literature encompassed randomized controlled trials, prospective cohort studies, observational registry-based analyses, pilot investigations, and retrospective audits. Four were randomized controlled trials evaluating liberalized clear-fluid fasting strategies or multimodal perioperative bundles [11,14,16,18]. Eight were prospective observational studies, including cohorts and interventional imaging protocols that assessed hemodynamic or ultrasound-derived parameters in relation to fasting duration [12,13,17,19,21,23,24,25]. Three large-scale retrospective or registry-based studies examined adherence to fasting guidelines and its association with hypotension and aspiration outcomes in real-world practice [4,10,22]. Two additional studies used audit or retrospective intervention designs to evaluate structured hydration policies and pre-induction intravenous fluid supplementation [15,20]. Adult data comprised randomized controlled trials, observational cohorts, interventional analyses, and one large multicenter implementation registry. Pediatric evidence was dominated by a large retrospective cohort, complemented by smaller observational and prospective physiological studies as well as audits. Several adult studies also incorporated bioimpedance to measure fluid status or echocardiographic/ultrasound methods to evaluate preload and its relationship with post-induction hypotension.

### 3.1. Adult Population

Evidence from studies in adult patients varied according to design, baseline risk, and assessment methods (Table 1). In this subgroup, actual fasting times for clear fluids often exceed guideline recommendations, with median durations of 4–6 h instead of the advised 2 h.

A retrospective cohort of 550 ASA I–III patients undergoing afternoon non-cardiac surgery evaluated the effect of morning intravenous fluids. Patients who received at least 1000 mL of fluid before surgery had a significantly lower incidence of post-induction hypotension, defined as a ≥20% reduction in systolic blood pressure, compared with those who remained fasting [15]. A prospective observational study in 130 ASA I–II patients undergoing ambulatory procedures found no independent association between fasting duration and hypotension, defined as mean arterial pressure < 70 mmHg or a >40% decrease following propofol induction, although hypotension remained frequent overall [12]. A randomized trial in 60 patients undergoing tympanoplasty compared ingestion of 200–1000 mL of oral rehydration solution two to three hours before anesthesia, but showed no significant differences in intraoperative mean arterial pressure or extracellular water distribution [18].

Other studies reported measurable fasting-related fluid deficits. A cohort of 115 adults assessed preoperative hydration with bioimpedance and demonstrated that abnormal intracellular-to-extracellular water ratios predicted hypotension, particularly at systolic blood pressure < 80 mmHg [13]. In 508 patients undergoing abdominal surgery, non-adherence to liberal fasting guidelines and longer actual fasting times correlated with more frequent post-induction hypotension [10]. Large-scale data confirmed that liberal fluid policies are feasible and safe. A multicenter quality improvement registry including 76,451 adults reported that allowing clear fluids until transfer to the operating room reduced median fasting times by three hours, improved comfort, and did not increase adverse gastric findings [4].

Interventional studies in higher-risk patients showed benefits from active strategies. In 107 high-risk non-cardiac surgery patients, a preventive bundle including optimized pre-induction fluid management significantly reduced hypotension compared with standard care [14]. In 57 patients undergoing coronary artery bypass grafting, preoperative carbohydrate loading combined with intraoperative omega-3 infusion reduced postoperative morbidity, illustrating the potential contribution of perioperative metabolic and fluid optimization strategies [11].

A systematic review of 12 studies identified advanced age, higher ASA class, emergency procedures, chronic ACE inhibitor or angiotensin receptor blocker use, and induction with propofol and opioids as consistent predictors of post-induction hypotension, with prolonged fasting reported as a modifiable contributor [26]. A narrative review similarly recognized fasting-related hypovolemia among the contributors to peri-induction hypotension [27].

Three of the included studies reported evidence on the risk of regurgitation and pulmonary aspiration. In a large cohort of over 76,000 cases, it was found that allowing clear fluids until transfer to the operating room, often resulting in fasting intervals shorter than two hours, reduced patient discomfort without increasing the incidence of regurgitation or aspiration events, which remained extremely rare and comparable to those observed with longer fasting durations [4]. Similarly, randomized and observational studies evaluating oral rehydration solutions or morning intravenous fluid supplementation did not report differences in aspiration-related complications between patients fasted for shorter versus longer intervals [15,18].

Among the included adult studies (Table 2), Morley et al. excluded patients on antihypertensive therapy, and induction was standardized with propofol infusion [12]. Iwayama et al. also excluded hypertensive patients; anesthesia was induced with propofol, fentanyl, and remifentanil, with sevoflurane for maintenance [18]. Siriopol et al. included patients on β-blockers and ACEI/ARB; induction consisted of propofol, opioids, and neuromuscular blockade [13]. Czajka et al. enrolled a large proportion of hypertensive patients (β-blockers, ACEI/ARB, diuretics) and induction was performed with propofol, opioids, and muscle relaxants [10]. In Marsman et al., details on both antihypertensive therapy and anesthetic induction were not reported [4]. Thomsen et al. studied high-risk patients, most on chronic antihypertensive treatment, and used titrated propofol with opioids and neuromuscular blockers [14]. Finally, Feguri et al. investigated cardiac patients on cardioprotective therapy (including β-blockers and statins); anesthesia was induced with etomidate, fentanyl, and pancuronium, and maintained with isoflurane [11].

Information on the duration of hypotension and advanced hemodynamic assessments was inconsistently reported. Zhang et al. measured both incidence and accumulated duration of hypotension within the first 15 min after induction, finding that prolonged fasting was associated with longer and more frequent episodes, which were mitigated by prophylactic morning intravenous fluids [15]. Czajka et al. distinguished post-induction from intraoperative hypotension and reported higher rates at both time points in patients with longer fasting and poor adherence to guidelines [10]. Thomsen et al. quantified hypotension severity using the area under the curve of MAP < 65 mmHg during the first 15 min after induction, providing an integrated measure of depth and duration [14].

The collected evidence consistently suggests that in adults, fasting longer than two hours is associated with a greater risk of hypovolemia and post-induction hypotension, whereas fasting for two hours or less is consistently safe with respect to regurgitation or aspiration and improves patient comfort, with emerging evidence suggesting better hemodynamic stability in selected groups.

### 3.2. Pediatric Population

Evidence from studies in pediatric patients was derived mainly from large retrospective cohorts, complemented by smaller prospective and physiological investigations (Table 3). According to the included studies, fasting intervals were generally longer than in adults, frequently exceeding 6 h and in some series extending beyond 10 h, despite recommendations for one to two hours.

The largest pediatric dataset was a retrospective cohort of 15,543 anesthetic events [22]. Arterial hypotension was defined as systolic blood pressure below the 2.5th percentile for age and sex, recorded every three minutes. Compared with children fasting less than four hours, those fasting longer showed progressively higher adjusted odds of hypotension OR: 1.27 for 4–6 h, 1.55 for 6–8 h, 1.33 for 12–14 h, and 1.28 for >14 h. The association was strongest during the surgical preparation epoch.

Smaller single-center studies provide complementary evidence. A prospective observational study in 100 infants younger than 36 months evaluated hemodynamic and metabolic endpoints [16]. Shorter fasting intervals were associated with more stable mean arterial pressure, lower serum ketone concentrations, and improved acid–base balance, indicating that prolonged fasting contributes to both circulatory and metabolic instability in this age group. A cross-sectional study of 50 children found a significant negative correlation between the duration of fasting for liquids and systolic blood pressure, with lower peri-induction values after more than 6 h of fluid abstinence; no aspiration or regurgitation events were recorded [17]. In a before-and-after study of 172 pediatric patients, mean fasting time for clear fluids was reduced following implementation of a structured “6-4-3-1” policy, and this improvement was associated with fewer episodes of peri-induction hypotension without any increase in regurgitation or aspiration events [23].

Audit data also highlight the practical link between fasting time and hypovolemia. Among 1820 pediatric patients undergoing anesthesia in a setting where a “one-hour clear fluid fasting” policy was implemented, the median actual fasting duration was 186 minutes, nearly three times longer than recommended. Children who experienced hypovolemia-related complications, such as difficult intravenous access or circulatory instability, had significantly longer fasting times (373 vs. 180 min, *p* < 0.001) [20]. A systematic review including eight pediatric studies further confirmed that prolonged fasting periods, particularly ≥12 h, were associated with dehydration, hypoglycemia, and hypotension after induction. Shortened fasting with carbohydrate-enriched clear fluids given one to two hours before anesthesia was consistently reported as safe, improving metabolic stability and perioperative comfort without increasing aspiration risk [28].

Several studies specifically addressed the risk of regurgitation and pulmonary aspiration in relation to shortened fasting. In the large retrospective cohort by Simpao et al., aspiration events were very rare and occurred at similar rates regardless of fasting duration, indicating that shorter fasting times did not increase risk [22]. Consistently, Hajian et al. and Thomasseau et al. observed no aspiration or regurgitation events with liberalized clear-fluid regimens, supporting the safety of allowing clear fluids up to one or two hours before anesthesia in children [17,23].

Among the pediatric studies, Hajian et al. described a standardized intravenous induction with propofol 2.5 mg/kg, atracurium 0.5 mg/kg, fentanyl 1–2 µg/kg, and midazolam 0.05 mg/kg, with maintenance using isoflurane (1–1.5%) in oxygen/nitrous oxide and neuromuscular block reversal with atropine (0.02 mg/kg) and neostigmine (0.07 mg/kg) [17]. Thomasseau et al. reported inhalational induction with sevoflurane and nitrous oxide, supplemented in some cases by propofol infusion, and maintenance with sevoflurane, nitrous oxide, and sufentanil [23]. In contrast, Simpao et al., Dennhardt et al., and Ricci et al. did not provide specific details on the anesthetic induction regimen [16,20,22].

Evidence in the pediatric patient population consistently shows that longer clear fluid fasting intervals are associated with arterial hypotension at anesthesia induction and postoperative metabolic derangement, whereas shorter intervals of one to two hours support stable perioperative hemodynamics without increasing aspiration risk.

### 3.3. Echocardiography and Ultrasound-Based Assessments

Evidence from echocardiographic and ultrasound-based studies was derived mainly from prospective mechanistic trials, complemented by larger observational cohorts (Table 4). Across the included studies, investigators applied transthoracic echocardiography, inferior vena cava measurements, and subclavian vein indices at different peri-induction intervals to evaluate preload and circulatory status.

In 146 ASA I–II adults, ultrasound of the subclavian vein after 12 h of fasting identified high collapsibility indices, and fluid therapy guided by this measure reduced the incidence of post-induction hypotension compared with standard practice [24]. Complementary findings were reported in a prospective study of 70 ASA I–II patients undergoing laparoscopic cholecystectomy, where midnight fasting for an average of 11.5 h was associated with significantly higher inferior vena cava collapsibility (27.7% vs. 17.8%) compared with a 4.3 h fasting interval, with collapsibility strongly correlating with fasting duration [21]. However, this study did not assess hypotension outcomes. Similarly, in a prospective trial of 40 patients fasted ≥ 12 h for gastrointestinal surgery, ultrasound-derived IVC variation after induction predicted fluid responsiveness and correlated with stroke volume index changes, while central venous pressure was not predictive [25].

Other imaging studies suggested that fasting alone does not always produce preload deficits. In 98 ASA I–III patients, transthoracic echocardiography performed before and after at least eight hours of fasting showed no clinically significant changes in stroke volume or preload indices [19]. In contrast, in 174 high-risk surgical patients, an interventional study incorporating continuous monitoring and echocardiography within a preventive bundle reduced the incidence of post-induction hypotension [14].

These findings indicate that echocardiography and ultrasound-derived parameters, applied at different peri-induction time points, effectively identified functional changes in patients after prolonged clear-fluid fasting compared with shorter fasting, and helped to recognize those at higher risk of peri-induction hypotension even when fasting duration alone was not predictive.

## 4. Discussion

This narrative review summarizes evidence on the relationship between duration of preoperative clear fluid fasting and arterial hypotension and echocardiographic changes at anesthesia induction. The findings indicate that prolonged fasting contributes to PIH mainly by aggravating hypovolemia and metabolic imbalance, although its role as an independent predictor is not consistent across all populations. In low-risk adults, fasting duration alone was not associated with hypotension once baseline physiology and anesthetic dose were considered. In contrast, larger cohorts in high-risk adults and pediatric series demonstrated that extended fasting independently increased both the incidence and duration of PIH. Bedside ultrasound studies further confirmed that prolonged fasting was associated with reduced intravascular volume, with abnormal IVC or subclavian collapsibility and altered body water distribution predicting hypotension despite standardized anesthetic regimens. Importantly, the aspiration risk that motivated restrictive fasting policies has not been supported by contemporary data, and ultrasound-based gastric assessments reinforce the safety of liberal clear-fluid policies.

The relevance of preoperative fasting extends beyond hemodynamic stability, encompassing significant metabolic implications. The updated 2023 ASA guidelines on preoperative fasting emphasize that prolonging fluid abstinence offers no additional protection against pulmonary aspiration, while it compromises patient comfort and may negatively affect hydration and metabolic balance [1]. The document explicitly supports the safety of clear fluids until two hours before anesthesia, underlining that unnecessary extension of fasting increases the risk of dehydration and metabolic stress, particularly in vulnerable patients such as the elderly, children, and individuals with comorbidities. These guidelines highlight that the rationale for liberalized fluid intake is not solely to prevent hypovolemia-related hypotension but also to mitigate the broader consequences of metabolic instability, including catabolism and impaired perioperative recovery. A review published in the World Journal of Diabetes underscores that excessive preoperative fasting adversely affects metabolic homeostasis [29]. The authors note that prolonged fasting induces insulin resistance, promotes lipolysis and ketogenesis, and alters glucose utilization, thereby increasing the metabolic burden during surgery. These changes are especially detrimental in diabetic patients but also impact non-diabetic populations, leading to suboptimal perioperative tolerance. The review further supports the role of preoperative carbohydrate-containing clear fluids in attenuating these metabolic disturbances, improving glycemic control, and reducing stress responses during the perioperative period. Together, these contributions broaden the perspective on preoperative fasting by framing it not only as a determinant of cardiovascular stability but also as a modifiable factor influencing perioperative metabolism. This dual hemodynamic and metabolic dimension strengthens the argument for liberalized clear fluid protocols and for considering carbohydrate-rich beverages as part of enhanced recovery pathways.

This review indicates that peri-induction hypotension (PIH) results from the interplay between anesthetic exposure, fasting-related hypovolemia, and patient vulnerability. In low-risk adults, Morley et al. found no independent association between fasting duration and the blood pressure drop during standardized propofol induction or the propofol dose required to reach BIS 50, with baseline MAP, weight, and sex emerging as stronger determinants [12]. In contrast, larger adult cohorts demonstrated that prolonged fasting, often coinciding with afternoon surgery, was independently associated with more frequent and longer PIH episodes; importantly, this risk was mitigated by pre-induction fluid therapy [10,14,15]. Studies employing bioimpedance and ultrasound monitoring further corroborated these findings: markers of hypohydration, such as abnormal intracellular-to-extracellular water ratios or increased IVC/subclavian collapsibility after >10–12 h fasting, predicted PIH despite standardized anesthetic regimens [13,19,21,24,25]. Patient characteristics also contributed to risk: advanced age, female sex, baseline low BP, chronic hypertension, and ACEI/ARB therapy consistently emerged as additional predictors [17,26]. Preventive interventions, including pre-induction volume expansion, vasopressor prophylaxis, and continuous echocardiographic monitoring, attenuate the hemodynamic consequences in high-risk adults [11,14]. Pediatric data reinforce these observations. Dennhardt et al. demonstrated that optimized fasting reduced ketone bodies and stabilized MAP [16], while Simpao et al. identified a clear fasting–dose response relationship with hypotension in more than 15,000 anesthetic events [22]. Other pediatric studies similarly showed that prolonged fasting exacerbates metabolic stress and circulatory instability [18,23]. These findings suggest that prolonged fasting is not uniformly an independent predictor of PIH: in carefully controlled, low-risk adult populations, fasting duration alone was not predictive once other variables were considered [12]. However, robust evidence from large adult cohorts and pediatric series indicates that extended fasting significantly increases PIH risk by aggravating intravascular depletion and metabolic imbalance, making it a clinically relevant and modifiable risk factor [10,14,15,22].

This review is based on a structured PubMed search strategy with multiple targeted keywords, which enabled the identification of a wide range of relevant studies, including randomized controlled trials, prospective cohorts, and registry-based analyses. This approach provided a comprehensive overview of both adult and pediatric populations and ensured that mechanistic investigations using ultrasound or bioimpedance were also captured, thereby enriching the interpretation of hemodynamic outcomes. The results obtained were consistent, showing a clear pattern of benefit from shorter fasting intervals across different study designs, which supports the validity of the search strategy. However, a limitation of this methodology is that the included studies employed heterogeneous definitions of hypotension and varied in their monitoring modalities, which prevented uniform comparison and limited the possibility of standardized outcome synthesis.

A key finding of this review is that ultrasound-based studies demonstrated measurable hypovolemia after prolonged fasting, including increased inferior vena cava collapsibility and reduced ventricular filling [14,19,21,24,25]. Several studies linked these changes to post-induction hypotension, providing a mechanistic explanation for the observed association between fasting duration and blood pressure instability, although some investigations reported no significant preload changes.

This review is limited by heterogeneity in definitions of hypotension, which ranged from mean arterial pressure below 60 mmHg to systolic arterial pressure below 100 mmHg in adults and age-adjusted thresholds in children. Study designs and surgical populations were variable, which restricts comparability. Most evidence came from elective surgery in patients without significant comorbidities, limiting applicability to higher-risk groups. Ultrasound was used in only a subset of studies, and techniques and thresholds for hypovolemia assessment were not standardized. However, the inclusion of both randomized trials and large observational datasets increases the generalizability of the findings. Consistent results across different study designs and patient groups support the reliability of the association between fasting duration and hypotension.

## 5. Conclusions

Prolonged preoperative fasting is not a universal independent predictor of peri-induction hypotension in low-risk adults, but evidence from large adult cohorts and pediatric populations demonstrates that extended fasting increases susceptibility to hypotension by aggravating hypovolemia and metabolic imbalance. Fasting duration should therefore be regarded as a clinically relevant and modifiable risk factor, particularly in high-risk and pediatric patients, where preventive strategies such as liberal clear fluid policies, pre-induction volume optimization, and tailored anesthetic management can mitigate the risk. Further studies should evaluate high-risk patients and standardize definitions and monitoring to optimize perioperative fluid management and to implement institutional protocols dedicated to shortening preoperative clear fluid fasting.

## Figures and Tables

**Table 1:** Adult population.

Reference	Design/*n*	Population and Intervention	Definition of Hypotension	Key Findings
Zhang et al., 2024 [15]	Retrospective cohort, *n* = 550	ASA I–III adults, afternoon non-cardiac surgery; ≥1000 mL morning IV fluids vs. fasting	≥20% ↓ in SBP	IV fluid group had significantly lower incidence of post-induction hypotension.
Morley et al., 2010 [12]	Prospective observational, *n* = 130	ASA I–II, ambulatory surgery, variable fasting	MAP < 70 mmHg or >40% ↓ after propofol	No independent association between fasting time and hypotension, but hypotension frequent.
Iwayama et al., 2014 [18]	RCT, *n* = 60	Tympanoplasty; 200–1000 mL ORS 2–3 h before anesthesia	MAP during 30–90 min after induction	No differences in MAP or extracellular water distribution between groups.
Siriopol et al., 2024 [13]	Cohort, *n* = 115	Elective surgery, preop bioimpedance	SBP < 80 mmHg or >20% MAP ↓	Abnormal I/E water ratio predicted hypotension.
Czajka et al., 2023 [10]	Cohort, *n* = 508	Abdominal surgery, fasting ≥ 8 h	MAP ≤ 65 mmHg	Longer fasting and non-adherence to guidelines correlated with more PIH.
Marsman et al., 2023 [4]	QI registry, *n* = 76,451	Adults, liberal policy vs. standard	Regurgitation/aspiration	Liberal policy ↓ fasting by 3 h, improved comfort, no ↑ aspiration.
Thomsen et al., 2025 [14]	Interventional bundle, n = 107	High-risk non-cardiac surgery	Area under MAP < 65 mmHg (15 min post-induction)	Preventive bundle (fluids, norepi, careful induction) minimized PIH.
Feguri et al., 2019 [11]	RCT, n = 57	CABG; CHO drink 2 h before + ω-3 PUFA infusion	Need for vasoactive drugs, POAF	CHO ↓ vasoactive use, infections; ω-3 ↓ POAF.

ASA: American Society of Anesthesiologists; CABG: coronary artery bypass grafting; CHO: carbohydrate; IV: intravenous; MAP: mean arterial pressure; ORS: oral rehydration solution; PIH: post-induction hypotension; POAF: postoperative atrial fibrillation; RCT: randomized controlled trial; SBP: systolic blood pressure; ↓: decrease; ↑: increase.

**Table 2:** Antihypertensive therapy and anesthetic induction in the adult population.

Study	Antihypertensive Therapy	Anesthetic Induction
Zhang et al., 2024 [15]	Included hypertensive patients; those with severe hypertension were excluded	Propofol with sufentanil, followed by sevoflurane and remifentanil maintenance
Morley et al., 2010 [12]	Patients with hypertension and antihypertensive use were excluded	Propofol infusion
Iwayama et al., 2014 [18]	Hypertensive patients were excluded	Propofol, fentanyl, and remifentanil for induction; sevoflurane for maintenance
Siriopol et al., 2024 [13]	Included patients on β-blockers and ACEI/ARB therapy	Propofol, opioids, and neuromuscular blockade
Czajka et al., 2023 [10]	46% of patients had hypertension; reported β-blocker, ACEI/ARB, and diuretic use	Propofol, opioids, and neuromuscular blockade, with regional techniques in ~10%
Thomsen et al., 2025 [14]	Majority of patients were hypertensive and on chronic therapy	Titrated propofol with opioids and neuromuscular blockers
Feguri et al., 2019 [11]	Cardiac patients, most receiving β-blockers and statins	Etomidate, fentanyl, and pancuronium for induction; isoflurane for maintenance

ACEI: angiotensin-converting enzyme inhibitor; ARB: angiotensin receptor blocker.

**Table 3:** Pediatric population.

Reference	Design/*n*	Population	Definition of Hypotension	Key Findings
Simpao et al., 2020 [22]	Retrospective cohort, *n* = 15,543	Children, elective surgery	SBP < 2.5th percentile for age/sex	Longer fasting associated with ↑ odds of hypotension, especially 4–8 h and >12 h.
Dennhardt et al., 2016 [16]	Prospective, *n* = 100 (50 + 50)	Infants < 36 months	MAP < 40 mmHg	Optimized fasting (6 h solids/2 h fluids) ↓ ketones, stabilized MAP.
Hajian et al., 2020 [17]	Cross-sectional, *n* = 50	Children, fasting vs. shorter	SBP peri-induction	Negative correlation between fasting > 6 h and SBP; no aspiration events.
Ricci et al., 2024 [20]	Audit, *n* = 1820	Pediatric hospital, “1-h clear fluid” protocol	Hypovolemia-related complications	Median fasting 186 min; longer fasting linked to circulatory instability, difficult IV access.
Thomasseau et al., 2021 [23]	Before–after, *n* = 172	Children, implementation of “6-4-3-1”	Hypotension episodes	Shorter fasting ↓ peri-induction hypotension, no aspiration.

MAP: mean arterial pressure; SBP: systolic blood pressure; ↓: decrease; ↑: increase.

**Table 4:** Echocardiography and ultrasound-based assessments.

Reference	Design/*n*	Method	Findings
Wang et al., 2024 [24]	Prospective, *n* = 146 + 124	Subclavian vein ultrasound; fluid therapy guided by SCVCI	SCVCI ≥ 45.4% predicted PIH; guided fluids ↓ PIH incidence.
Shivhare et al., 2023 [21]	Prospective, *n* = 70	IVC collapsibility, laparoscopic cholecystectomy	Midnight fasting (~11.5 h) → higher IVC collapsibility vs. 4.3 h fast; correlated with fasting time.
Zhang X et al., 2016 [25]	Prospective, *n* = 40	IVC variation vs. stroke volume index	IVC variation predicted fluid responsiveness; CVP not predictive.
Muller et al., 2014 [19]	Prospective, *n* = 98	TTE before/after ≥8 h fasting	No significant preload or SV changes overall.
Thomsen et al., 2025 [14]	Interventional, *n* = 174	Continuous echo monitoring in bundle	Reduced PIH vs. standard care.

CVP: central venous pressure; IVC: inferior vena cava; PIH: post-induction hypotension; SCVCI: subclavian vein collapsibility index; SV: stroke volume; TTE: transthoracic echocardiography; ↓: decrease; ↑: increase.

## Data Availability

This study did not generate new data. All data discussed in this review are available in the published article retrieved through PubMed and cited in the reference list.

## Abbreviations

The following abbreviations are used in this manuscript:

ASA	American Society of Anesthesiologists
ESAIC	European Society of Anaesthesiology and Intensive Care
IVC	Inferior Vena Cava
MAP	Mean Arterial Pressure
OR	Odds Ratio
SAP	Systolic Arterial Pressure
